# Clinicopathological features of laterally spreading colorectal tumors and their association with advanced histology and invasiveness: An experience from Honam province of South Korea: A Honam Association for the Study of Intestinal Diseases (HASID)

**DOI:** 10.1371/journal.pone.0184205

**Published:** 2017-10-04

**Authors:** Dae-Seong Myung, Sun-Seog Kweon, Jun Lee, Ik-Sang Shin, Sang-Wook Kim, Geom-Seog Seo, Hyun-Soo Kim, Young-Eun Joo

**Affiliations:** 1 Department of Internal Medicine, Chonnam National University Medical School, Gwangju, Korea; 2 Department of Preventive Medicine, Chonnam National University Medical School, Gwangju, Korea; 3 Department of Internal Medicine, Chosun University College of Medicine, Gwangju, Korea; 4 Department of Internal Medicine, Chonbuk National University Medical School, Jeonju, Korea; 5 Department of Internal Medicine, Wonkwang University College of Medicine, Iksan, Korea; National Cancer Center, JAPAN

## Abstract

**Background and aims:**

Laterally spreading colorectal tumors (LSTs) are divided into four subtypes, including homogenous (HG), nodular mixed (NM), flat elevated (FE), and pseudo-depressed (PD), based on their different endoscopic morphologies. The aim of this study was to investigate the clinicopathological significance of LST subtypes and their association with advanced histology.

**Methods:**

We investigated the medical records of consecutive patients with LST who initially underwent endoscopic resection at five university hospitals in Honam province of South Korea between January 2012 and December 2013. A total of 566LST lesions removed via endoscopic procedures were collected retrospectively for data analysis.

**Results:**

The PD, FE, and NM subtypes were more common in the distal colon and the HG subtype in the proximal colon. The PD subtype had the biggest tumor size, followed by the NM subtype. The frequency of adenomatous pit pattern was significantly higher in the HG, NM, and FE subtypes than in the PD subtype. In contrast, the frequency of cancerous pit pattern was significantly higher in the PD subtype than in the other three subtypes. The rate of advanced histology (high-grade dysplasia or carcinoma) among the LSTs was 36.0%. The risk of advanced histology increased in the distal colon compared with the proximal colon. The PD subtype had the highest incidence of villous component, advanced histology,submucosal invasion, and postprocedure perforation among the four subtypes. The distal colon as tumor site, larger tumor size, PD subtype, and villous component were associated with a statistically significant increased risk of advanced histology.

**Conclusion:**

Our results indicate that the location, size, endoscopic subtype, and histologic component of the LSTs are associated with an increased risk of advanced histology. Therefore, these clinicopathological parameters may be useful in selecting therapeutic strategies in the clinical setting.

## Introduction

Colorectal cancer is one of the most common malignancies and is still one of the major causes of cancer-related mortality worldwide. Its incidence and mortality have shown a decreasing trend over the last several decades. The application of colorectal cancer screening program has contributed to this trend. Colonoscopy is an effective colorectal cancer screening and prevention modality as evidenced by a decline in both incidence and mortality of colorectal cancer[1–3].

Laterally spreading colorectal tumors (LSTs) are non-polypoid neoplastic lesions with a diameter of at least 10 mm, which typically extend laterally rather than vertically along the interior luminal wall[4–6].Previous studies have shown that the prevalence of LST was 0.8–5.2% in asymptomatic and average-risk populations that underwent screening colonoscopy[4–6]. However, because it is difficult to detect superficial flat neoplastic lesions such as LSTs with optical colonoscopy, LSTs are of special interest of endoscopists and constitute a major target for colorectal cancer screening. Recently, wider applications of LSTs in screening colonoscopy have been reported with advances in endoscopic technology and operator skills [7–13].

According to their surface morphological features, LSTs are usually classified into two types and four subtypes: the granular type (LST-G), including homogeneous (HG) and nodular mixed (NM) subtypes and the non-granular type (LST-NG), including flat elevated(FE) and pseudo-depressed (PD) subtypes[4–6]. The molecular characteristics of the LST-G and LST-NG types differ[14, 15]. Further, the clinicopathological characteristics of the four subtypes vary according to different populations[7–13].

Histologically, 90% of LSTs are adenomas [4–6]. Contrary to other non-polypoid neoplastic lesions, the frequency of LSTs with invasive carcinoma is lower than that of polypoid lesions with a similar size[4–13]. Therefore, LSTs are usually removed via endoscopic procedures, such as endoscopic mucosal resection (EMR), endoscopic mucosal resection with precutting(EMRP), and endoscopic submucosal dissection (ESD)[16–27]. However, more than 30% of LSTs contain advanced histology such as high grade dysplasia and adenocarcinoma [4–13]and the rate of advanced histology in LST varies in accordance with tumor size and subtype[16–27]. Previous studies have shown that LST-NG type has an invasive nature with malignancy rate higher than that of LST-G and large tumors of PD and NM subtypes have a higher malignant potential[4–13, 16–27].Therefore, to avoid either incomplete endoscopic resection or unnecessary surgery in patients with LST, it is clinically important to predict advanced histology and invasiveness for selecting the appropriate therapeutic plan of LSTs. The aim of this study was to evaluate the endoscopic, morphologic, and clinicopathological parameters of LSTs and their association with advanced histology in a Korean population.

## Materials and methods

### Patients

The Honam Association for the Study of Intestinal Diseases (HASID) is a collaborative initiative developed to collect retrospective data of patients undergoing endoscopic resections of LSTs. This study evaluated consecutive patients who initially underwent endoscopic resection for LSTs in five university hospitals at Honam province of South Korea between January 2012 and December 2013.One physician at each hospital was responsible for data collection, and the completeness of the data collection was monitored by one of the authors (Y.E.J.). A total of 837 patients underwent endoscopic procedures, such as EMR, EMRP, and ESD in an attempt to remove an additional LST lesion (220 patients from Chonnam National University Hwasun Hospital,204 patients from Chonnam National University Hospital, 202 patients from Chonbuk National University Hospital, 112 patients from Chosun University Hospital, 99 patients from Wonkwang University Hospital). One lesion was randomly selected from the multiple LST lesions. Surgery was recommended for lesions with a non-lifting sign; difficult approach of endoscopic therapy, in terms of the size and location; margin positivity; extensive invasive lesions(cancer cell invasion>1000μm from the muscularis mucosa); and complications after endoscopic procedures. Furthermore, we excluded 271 patients owing to the lack of complete clinicopathological data (220patients), surgery(eight patients), and non-neoplastic lesions, such as hyperplastic polyp and chronic colitis (43patients). Finally, a total of 566 LST lesions were statistically analyzed retrospectively for variable clinicopathological characteristics, including endoscopic subtype, size, location, pit pattern analysis, and histopathology (S1 File). The study was performed in accordance with the ethical principles of the Declaration of Helsinki and was approved by the Institutional Review Board of each hospital; written informed consent was obtained from all patients prior to the endoscopic procedures.

### Endoscopic criteria of LST

**Fig 1:**
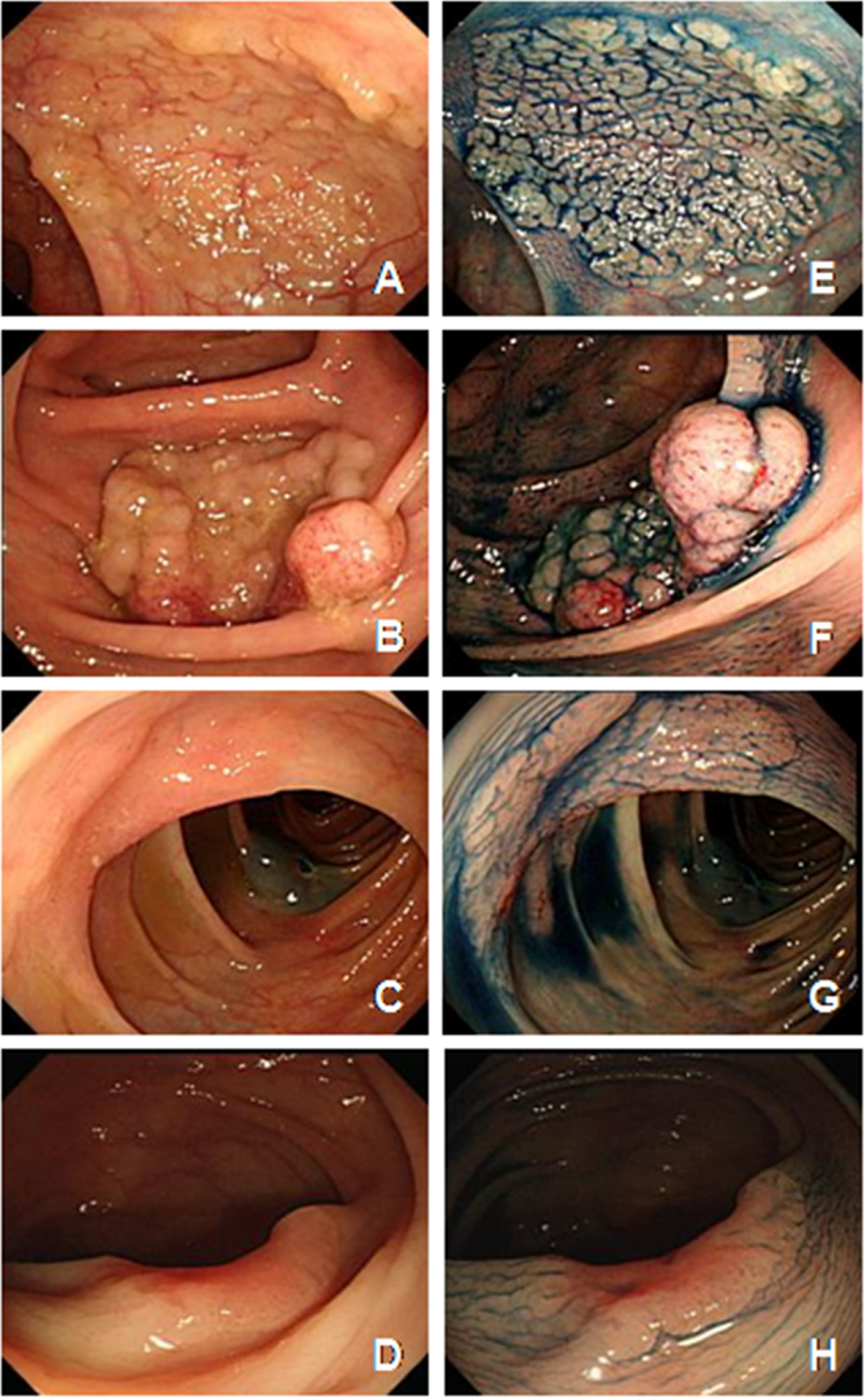
**Endoscopic (A-D) and chromoscopic findings (E-H)with 0.4% indigo-carmine dye spraying of laterally spreading tumors (LSTs).** A, E. LST-Granular-Homogenous (LST-G-HG). B, F. LST-Granular-Nodular mixed (LST-G-NM). C,G. LST-Non-granular-Flat elevated (LST-NG-FE). D, H. LST-Non-granular-Psueodepressed (LST-NG-PD).

All patients were examined using video colonoscopes (Olympus CF-240I or CF-H260; Olympus, Tokyo, Japan). LSTs were defined as lesions> 10mm in diameter with a low vertical axis extending laterally along the colonic luminal wall. LSTs were categorized into two types based on their endoscopic findings: either LST-G, which has the conglomerates of even or uneven nodules or granules, forming a flat broad-based lesion including the HG and NM subtypes, or LST-NG, which has a flat smooth surface appearance without nodules or granules including the FE and PD subtypes(Fig 1) [4–6]. When LSTs were detected via conventional colonoscopy, the colonoscopists used narrow band imaging (NBI) or dye-spray chromoendoscopy with indigo carmine to enhance the lesion surface details, such as pit pattern, presence of large nodule, depression, and chicken skin mucosa[19]. The pit pattern of their lesions was especially evaluated retrospectively by two observers (D.S.M. and Y.E.J.) while analyzing the conventional colonoscopy, NBI, or chromoendoscopic images. Among the 566 LST lesions, a consensus was reached in 433 LST lesions by an interobserver agreement. The pit pattern was divided into six groups according to Kudo’s classification system: types I, II, IIIs, IIIL, IV, and V. An invasive pit pattern is characterized by an irregular and distorted epithelial crest or pit pattern loss[28, 29]. The location of the LST was categorized as follows: distal colon (rectosigmoid colon and descending colon) and proximal colon (transverse colon, ascending colon, and cecum).

### Histopathological analysis of LST

For the histopathological analysis, all resected specimens were immediately fixed in a 10% buffered formalin solution and examined histologically using hematoxylin and eosin staining. All resected specimens were examined by experienced gastrointestinal pathologists. The histopathological diagnosis was determined in accordance with the World Health Organization criteria[30]. Intraepithelial neoplasm was defined as either low-grade dysplasia or high-grade dysplasia. Carcinoma in situ was included under high-grade intraepithelial neoplasm. High-grade intraepithelial neoplasm and submucosal invasive carcinoma were defined as an advanced histology[31].

### Statistical analysis

Statistical analyses were performed using the Statistical Package for the Social Sciences (Version 18.0; SPSS, Chicago, USA).The descriptive analysis included proportions for categorical data as well as means ± standard deviations (SDs) for continuous data. Differences were analyzed using the chi-square test, Student’s *t*-test and analysis of Variance test, as appropriately. Ordered variables, such as pit pattern and histologic grade, were compared with Kruskal Wallis H test. Furthermore, we used the binary logistic regression model to identify the risk factors of advanced histology of the LSTs. Age at diagnosis was adjusted in the basic model, and additional potential confounders, such as sex, smoking, and aspirin/NSAID use history, were adjusted in the full model. For all tests, a *P*-value of<0.05 was considered statistically significant.

### Ethics statement

This study was approved by the Institutional Review Board of Chonnam National University Hwasun Hospital(2013–149), the Institutional Review Board of Chosun University Hospital(2014-02-005), the Institutional Review Board of Chonbuk National University Hospital (2014-01-005-002) and the Institutional Review Board of Wonkwang University Hospital(WKUH 201401-HRE-010).(S2 File) A written informed consent was obtained from each participant prior to endoscopy. All participants gave written consent of their information to be stored in the hospital database and used for research.

## Results

### Demographic data of the patients with LST

The demographic data of the patients with LST are summarized in Table 1. The mean age of the patients enrolled in the study was 65.4±9.7 years with a range from 31.0 to 90.0. This study group comprised 359 men and 207 women. The male-to-female sex ratio was 1.7:1. Among the 566 LSTs, 376 were LST-G, including 141 HG (24.9%) and 235 NM subtypes (41.5%),and 190 were LST-NG, including 156 FE (27.6%) and 34 PD subtypes (6.0%). The mean size of the LSTs was 24.2±13.4 mm (range, 10.0–80.0). Among the LSTs, 266 (47.0%) were localized in the proximal colon and 300 (53.0%) in the distal colon. According to the classification of Kudo’s pit pattern, 75 (13.3%) lesions were type I, 25 (4.4%) were type II, 99 (17.5%) were type IIIs, 164 (29.0%) were type IIIL, 19 (3.4%) were type IV, 33 (5.8%) were type Vi, and 18 (3.2%) were type Vn. Regarding the histologic components, 428 (75.6%) LSTs had tubular, 110 (19.4%) had tubulovillous, and 28 (4.9%) had villous components. Regarding the histologic grades, 362 (64.0%) were low-grade dysplasias, 115 (20.3%) were high-grade dysplasias, and 89 (15.7%) were adenocarcinomas with 60 mucosal invasion (10.6%) and 29 submucosal invasion (5.1%). The rate of the LSTs with advanced histology (high-grade dysplasia and adenocarcinoma, including carcinoma in situ or submucosal cancer) was 36.0% (204/566). The LSTs were removed via EMR (285, 50.4%), EMRP (83, 14.7%), ESD (193, 34.1%),or surgery (5, 0.8%). The en bloc, piecemeal resection, and surgery rates were 84.5% (478/566), 14.7% (78/566), and 0.8% (5/566), respectively. The postprocedure bleeding rate was 8.3% (47/566), and the perforation rate was 1.6% (9/566).

**Table 1:** Clinicopathological parameters of the patients with colorectal laterally spreading tumors.

Parameter			n = 566 (%)
Age (years)	Mean±SD (range)		65.4±9.7 (31.0–90.0)
Gender	Male/Female		359/207 (63.4/36.6)
Smoking status	Non-smoker/current or ex-smoker		426/140 (75.3/24.7)
Alcohol drinking	No/Yes		394/172 (69.6/30.4)
BMI (Kg/m^2^)	Mean±SD (range)		24.1±13.4 (15.5–38.7)
Endoscopic type	Granular		376 (66.4)
	Homogenous		141 (24.9)
	Nodular mixed		235 (41.5)
	Non-granular		190 (33.6)
	Flat elevated		156 (27.6)
	Pseudo-depressed		34 (6.0)
Size (mm)	Mean±SD (range)		24.2±13.4 (10.0–80.0)
Location	Proximal colon		266 (47.0)
	Distal colon		300 (53.0)
Pit pattern (n = 433)	Non-neoplastic (Type I/II)		75/25 (13.3/4.4)
	Adenomatous (Type IIIs/IIIL/IV)		99/164/19 (17.5/29.0/3.4)
	Cancerous (Type Vi/Vn)		33/18 (5.8/3.2)
Histologic component	Tubular		428 (75.6)
	Tubulovillous		110 (19.4)
	Villous		28 (4.9)
Histologic grade	Low grade dysplasia		362 (64.0)
	High grade dysplasia		115 (20.3)
	Adenocarcinoma Mucosal invasion Submucosal invasion		89 (15.7) 60 (10.6) 29 (5.1)
Treatment	EMR		285 (50.4)
	EMRP		83 (14.7)
	ESD		193 (34.1)
	Surgery		5 (0.8)
Resection method	En bloc resection		478 (84.5)
	Piecemeal resection		78 (14.7)
	Surgery		5 (0.8)
Complication	Bleeding	No	519 (91.7)
		Yes	47 (8.3)
	Perforation	No	557 (98.4)
		Yes	9 (1.6)

SD, standard deviation; BMI, body mass index; EMR, endoscopic mucosal resection; EMRP, endoscopic mucosal resection with precutting; ESD, endoscopic submucosal dissection

### Comparison of clinicopathological parameters according to LST subtypes

Table 2 shows the clinicopathological characteristics of the LST subtypes according to endoscopic appearance. The size of the LST was significantly different by subtypes (*P*< 0.001); the PD subtype had the biggest tumor size (mean±SD, 30.3±15.5mm), followed by the NM subtype (mean±SD, 27.7±13.2mm). The BMI of the patients with the FE and PD subtypes was higher than that of the patients with the HG and NM subtypes (*P* = 0.014). The location of the LSTs differed significantly by subtypes (*P*< 0.001). The HG subtype was more common in the proximal colon, whereas the NM, FE, and PD subtypes were more common in the distal colon. In the pit pattern analysis of the LSTs, we found that 64.7% of the HG subtype, 63.1% of the NM subtype, and 77.1% of the FE subtype had an adenomatous pit pattern (IIIs/IIIL/IV). In contrast, the frequency of cancerous pit pattern (Vi/Vn) was significantly higher in the PD subtype than in the other three subtypes (*P*< 0.001). The incidence rate of advanced histology was 73.5%, 46.8%, 25.6%, and 21.3% in the PD, NM, FE, and HG subtypes, respectively. Also, the incidence rate of submucosal adenocarcinoma was 11.7%, 8.1%, 2.8%, and 1.3% in the PD, NM, HG, and FE subtypes, respectively. The PD subtype had the highest incidence of villous component, high-grade dysplasia, and adenocarcinoma with mucosal and submucosal invasion among the four subtypes (*P*< 0.001). In the endoscopic treatment of the LSTs, ESD was performed more commonly in the PD and NM subtypes than in the HG and FE subtypes (*P*< 0.001). Postprocedure perforation, chicken skin mucosa, and depressed lesion were significantly more common in the PD subtype than in the other three subtypes (*P*< 0.002, *P* = 0.001, and *P*< 0.001, respectively). There were no statistically significant differences between the four subtypes in terms of resection method and postprocedure bleeding (*P* = 0.780 and *P* = 0.147, respectively). When we compared the differences according to macroscopic types, granular and non-granular type, the significance was disappeared for the variables of location, histologic grade, postprocedure perforation, and chicken skin mucosa (Table 2).

**Table 2:** Comparison of clinicopathological parameters of the LST subtypes according to endoscopic appearance.

Parameter	Macroscopic type				Differences between subtypes (*P*-value)	Differences between macroscopic types (*P*-value)
	Granular type (n = 376)		Non-granular type (n = 190)			
	HG (n = 141)	NM (n = 235)	FE (n = 156)	PD (n = 34)		
Age (years)	65.5±10.0	65.0±10.4	66.2±8.8	64.0±9.1	0.623	0.439
Size (mm)	21.2±14.9	27.7±13.2	20.2±9.3	30.3±15.5	<0.001	0.007
BMI (Kg/m^2^)	23.9±3.1	23.6±2.9	24.7±3.5	24.4±3.1	0.014	0.002
Size (mm)					<0.001	0.026
<20	69 (48.9)	54 (23.0)	72 (46.2)	9 (26.5)		
20–30	53 (37.6)	105 (44.7)	66 (42.3)	10 (29.4)		
>30	19 (13.5)	76 (32.3)	18 (11.5)	15 (44.1)		
Location					<0.001	0.139
Proximal colon	84 (59.6)	101 (43.0)	69 (44.2)	11 (32.4)		
Distal colon	57 (40.4)	134 (57.0)	87 (55.8)	23 (67.6)		
Pit pattern (n = 433)					<0.001	0.028
I/II	39 (32.8)	39 (22.2)	20 (19.0)	2 (5.9)		
IIIs/IIIL/IV	77 (64.7)	111 (63.1)	81 (77.1)	14 (41.2)		
Vi/Vn	3 (2.5)	26 (14.8)	4 (3.8)	18 (52.9)		
Histologic component					<0.001	<0.001
Tubular	114 (80.9)	141 (60.0)	146 (93.6)	26 (76.4)		
Tubulovillous	20 (14.2)	79 (33.6)	8 (5.1)	4 (11.8)		
Villous	7 (5.0)	15 (6.4)	2 (1.3)	4 (11.8)		
Histologic grade					<0.001	0.623
Low grade dysplasia	111 (78.7)	125 (53.2)	116 (74.4)	9 (26.5)		
High grade dysplasia	20 (14.2)	58 (24.7).	28 (17.9)	10 (29.4)		
Adenocarcinoma	10 (7.1)	52 (22.1)	12 (7.7)	15 (44.1)		
Mucosal invasion	6 (4.3)	33 (14.0)	10 (6.4)	11(32.4)		
Submucosal invasion	4 (2.8)	19 (8.1)	2 (1.3)	4 (11.7)		
Treatment					<0.001	<0.001
EMR	92 (65.3)	97 (41.3)	87 (55.8)	9 (26.5)		
EMRP	16 (11.3)	23 (9.8)	37 (23.7)	7 (20.6)		
ESD	30 (21.3)	113 (48.1)	32 (20.5)	18 (52.9)		
Surgery	3 (2.1)	2 (0.8)	0 (0.0)	0 (0.0)		
Resection method					0.780	0.527
En-bloc	119 (84.4)	201 (85.5)	132 (84.6)	26 (76.5)		
Piecemeal	19 (13.5)	32 (13.6)	24 (15.4)	8 (23.5)		
Surgery	3 (2.1)	2 (0.8)	0 (0.0)	0 (0.0)		
Perforation (-)	137 (97.1)	234 (99.6)	154 (98.7)	32 (94.1)	0.002	0.320
Bleeding (-)	134 (95.0)	219 (93.2)	136 (87.2)	30 (88.2)	0.147	0.052
Chicken skin mucosa (-)	134 (95.0)	198 (84.3)	120 (76.9)	24 (72.7)	0.001	0.270
Depressed lesion (-)	137 (97.1)	212 (90.2)	141 (90.4)	5 (15.2)	<0.001	<0.001

HG, homogenous; NM, nodular mixed; FE, flat elevated; PD, pseudodepressed; BMI, body mass index; EMR, endoscopic mucosal resection; EMRP, endoscopic mucosal resection with precutting; ESD, endoscopic submucosal dissection

### Comparison of clinicopathological parameters of LST according to histology

Table 3 shows the clinicopathological characteristics of the LSTs according to histology. The patients with LSTs with advanced histology had a bigger mean tumor size and higher BMI than those with LSTs with non-advanced histology (*P*< 0.001 and *P* = 0.034, respectively). The LSTs with advanced histology were more common in the distal colon and the LSTs with non-advanced histology in the proximal colon. The frequency of cancerous pit pattern (Vi/Vn) was significantly higher in the LSTs with advanced histology than in the LSTs with non-advanced histology (*P*< 0.001). The incidence of villous component in the LSTs with advanced histology was higher than that in the LSTs with non-advanced histology (*P*< 0.001). ESD was performed more commonly in the LSTs with advanced histology than in the LSTs with non-advanced histology (*P*< 0.001). Chicken skin mucosa and depressed lesion were more common in the LSTs with advanced histology than in the LSTs with non-advanced histology (*P* = 0.001 and *P* = 0.002, respectively). There were no statistically significant differences between the LSTs with advanced histology and with non-advanced histology in terms of postprocedure perforation and bleeding (*P* = 0.753 and *P* = 0.307, respectively).

**Table 3:** Comparison of clinicopathological parameters of the LST according to histology.

Parameter	Non-advanced histology		Advanced histology	*P*-value
Number	362 (64.0)		204 (36.0)	
Age (years)	64.4 ±10.2		66.4±9.3	0.076
Mean size (mm)	21.0±10.7		29.6±15.6	<0.001
BMI (Kg/m^2^)	24.3±3.3		23.7±2.9	0.034
Size (mm)				<0.001
<20	163 (45.0)		40 (19.6)	
20–30	140 (38.7)		93 (45.6)	
>30	59 (16.3)		71 (34.8)	
Location				<0.001
Proximal colon	197 (54.4)		69 (33.8)	
Distal colon	165 (45.6)		135 (66.2)	
Pit pattern (n = 433)				<0.001
I/II	73 (26.3)		27 (17.4)	
IIIs/IIIL/IV	202(72.7)		80 (51.6)	
Vi/Vn	3 (1.1)		48 (31.0)	
Histologic component				<0.001
Tubular	301 (83.1)		127 (62.3)	
Tubulovillous	53 (14.6)		57 (27.9)	
Villous	8 (2.2)		20 (9.8)	
Histologic grade				
Low-grade dysplasia	362 (100.0)		0 (0.0)	
High grade dysplasia	0 (0.0)		115 (56.4)	
Adenocarcinoma	0 (0.0)		89 (43.6)	
Treatment				<0.001
EMR	221 (61.0)		64 (31.5)	
EMRP	50 (13.8)		32 (15.8)	
ESD	90 (24.9)		104 (51.2)	
Surgery	1 (0.3)		4 (1.5)	
Resection method				<0.001
En-bloc	320 (88.4)		158 (77.5)	
Piecemeal	41 (10.8)		42 (20.6)	
Surgery	1 (0.8)		4 (1.9)	
Perforation (-)	355 (98.1)		202 (99.0)	0.753
Bleeding (-)	336 (92.8)		183 (89.7)	0.307
Chicken skin mucosa (-)	312 (86.2)		164 (80.4)	0.001
Depressed lesion (-)	326 (90.1)		169 (82.8)	0.002

BMI, body mass index; EMR, endoscopic mucosal resection; EMRP, endoscopic mucosal resection with precutting; ESD, endoscopic submucosal dissection

### Risk of advanced histology according to clinicopathological parameters of the LSTs

The risk of advanced histology among the LSTs increased in the distal colon compared with the proximal colon [OR = 2.31, 95% CI (1.62–3.30)]. Tumor size, endoscopic subtypes, and histologic components were also associated with a statistically significant increased risk of advanced histology [OR = 2.73, 95% CI (1.76–4.22) for 20–30 mm *vs*. under 20 mm; OR = 5.17, 95% CI (3.16–8.48) for over 30 mm *vs*. under 20 mm; OR = 3.33, 95% CI (2.06–5.38) for NM *vs*. HG subtypes; OR = 10.40, 95% CI (4.35–24.85) for PD *vs*. HG subtypes; OR = 2.57, 95% CI (1.67–3.95) for tubulovillous *vs*. tubular components; OR = 6.05, 95% CI (2.59–14.14) for villous *vs*. tubular components]. The significant association between advanced histology and the parameters persisted after adjustments for additional confounders in the full model. The LSTs with chicken skin mucosa [OR = 2.51, 95% CI (1.45–4.36)] and depressed lesion [OR = 2.27, 95% CI (1.35–3.83)] were associated with an increased risk of advanced histology; however, no significant association was found after full adjustments (Table 4).

**Table 4:** Risk of advanced histology according to clinicopathological parameters in colorectal laterally spreading tumors.

	Age-adjusted		Fully-adjusted	
	OR (95% CI)	*P*-value	OR (95% CI)	*P*-value
Male Sex	0.88 (0.62–1.26)	0.485	1.14 (0.71–1.83)	0.040
Ex-/current smoker	1.22 (0.82–1.81)	0.325	1.44 (0.84–2.45)	0.183
Alcohol drinking	1.07 (0.74–1.57)	0.708	1.13 (0.69–1.86)	0.637
Aspirin/NSAID user	1.00 (0.61–1.67)	0.982	1.35 (0.75–2.43)	0.315
Location				
Proximal colon	1.0 (ref.)		1.0 (ref)	
Distal colon	2.31 (1.62–3.30)	<0.001	2.08 (1.39–3.11)	<0.001
Size (mm)				
<20	1.0 (ref.)		1.0 (ref)	
20–30	2.73 (1.76–4.22)	<0.001	2.49 (1.53–4.06)	<0.001
>30	5.17 (3.16–8.48)	<0.001	3.20 (1.81–5.65)	<0.001
Endoscopic subtype				
HG	1.0 (ref.)		1.0 (ref)	
NM	3.33 (2.06–5.38)	<0.001	2.12 (1.24–3.61)	0.006
FE	1.26 (0.73–2.17)	0.399	1.33 (0.73–2.44)	0.355
PD	10.4 (4.35–24.9)	<0.001	7.03 (2.30–21.5)	0.001
Histologic component				
Tubular	1.0 (ref.)		1.0 (ref)	
Tubulovillous	2.57 (1.67–3.95)	<0.001	1.97 (1.16–3.35)	0.012
Villous	6.05 (2.59–14.13)	<0.001	3.85 (1.52–9.75)	0.005
Chicken-skin mucosa (+)	2.51 (1.45–4.36)	0.001	1.87 (0.99–3.48)	0.050
Depressed lesion (+)	2.27 (1.35–3.83)	0.002	1.07 (0.50–2.30)	0.865

NSAID, non-steroidal anti-inflammatory drug; HG, homogenous; NM, nodular mixed; FE, flat elevated; PD, pseudodepressed

## Discussion

Here, we retrospectively evaluated a large number of patients with LSTs treated with endoscopic resection at five university hospitals in Honam province of South Korea, and investigated the clinicopathological significance of the LST subtypes and their association with advanced histology.

Our study showed a higher incidence in men and patients aged≥60 years (mean age, 65.4 years), which was similar to those of previous studies in Romanian, Korean, Italian, Japanese and Chinese populations [7–13]. LSTs are previously reported to be more frequent in the proximal colon in Japan and Italy [6, 9] in distal colon in China [13]. In addition, LST-NG was more frequent in the proximal colon in Japan (19). LST-G was more frequent in distal colon in China and Japan [13, 19], and in proximal colon in Italy [9].However, our study showed no significant difference in the proximal or distal location of the LSTs and LST-NG was more common in the distal colon. There are possible explanations for these inconsistent results between our and other reports. First, different populations have distinct clinical characteristics of LSTs. Second, the sample size of our and other reports was variable, thus selection biases may be unavoidable.

Generally, LSTs is less invasive than that of other polypoid lesions with a similar size[4–13]. Considering the relative benign nature of LSTs, many endoscopists have attempted to use endoscopic resection as the first-line treatment[16–18]. However, LSTs involve heterogenous groups, including four distinct endoscopic morphologies and different surface characteristics, such as pit pattern and regional colonic mucosa[4–13]. These lesions have a malignant potential according to their different clinical parameters. According to previous studies, the incidence rate of LSTs with advanced histology, such as high-grade dysplasia and adenocarcinoma, ranged from 20.9% to 33.8%[4–13]. Our study showed that the rate of the LSTs with advanced histology was 36.0%. Among the advanced histology, the incidence rate of adenocarcinoma was 15.7%, and submucosal adenocarcinoma was 5.1%.Therefore, it is essential to recognize the features of LSTs that might predict a higher incidence of cancer with deep submucosal invasion.

Next, we compared the clinicopathological parameters according to the LST subtypes. In our study, the PD subtype had the biggest tumor size, followed by the NM subtype. The PD, FE, and NM subtypes were more common in the distal colon except the HG subtype. In the pit pattern analysis considering as an indicator of submucosal invasion, we found that the frequency of adenomatous pit pattern (IIIs/IIIL/IV) was significantly higher in the HG, NM, and FE subtypes than in the PD subtype. In contrast, the frequency of cancerous pit pattern (Vi/Vn) was significantly higher in the PD subtype than in the other three subtypes. Previous study showed that adenomatous pit pattern(IIIL/IV) is a dominant pit pattern in HG and FE subtypes and cancerous pit pattern(Vi/Vn) is a predominant pattern in NM and PD subtypes [13]. Also, the incidence rate of advanced histology and submucosal adenocarcinoma was significantly higher in the PD than in the other three subtypes. Previously, advanced histology was reported frequently in the NM and PD subtypes, and the proportion of submucosal invasion also increased in the PD subtype in accordance with our results[4–13]. In our study, the PD subtype was associated more frequently with larger tumor size, distal location, cancerous pit pattern, advanced histology, and submucosal invasion than the other three subtypes.

The size, location, pit pattern, and subtype of LSTs are well-known predictors of advanced histology [4–13]. Thus, we compared the clinicopathological parameters of the LSTs according to histologic grade. In our study, larger tumor size, distal location, cancerous pit pattern, villous component, chicken skin mucosa, and depressed lesion were more common in the LSTs with advanced histology than in the LSTs with non-advanced histology, indicating that these maybe predictive markers of advanced histology in LSTs.

LSTs are usually treated via an endoscopic approach, including EMR, EMRP, and ESD, according to the size and location of the LSTs and operator’s discretion [16–18]. In our study, the LSTs were removed via EMR (50.4%), ESD (34.1%),and EMRP (14.7%). The rate of *en bloc* resection was 84.5%. ESD has the advantage of enabling precise histologic evaluation of resected specimens and disadvantage with a higher rate of complications, such as bleeding and perforation, and requires a long procedure duration [16–18]. Previous studies recommended that LST-NGs larger than 20 mm and LST-Gs larger than 30 mm should be managed using ESD with *en bloc* resection[19, 32].According to our study results, ESD allowing more accurate histologic evaluation with *en bloc* resection and reducing recurrence rate was performed more commonly for PD and NM subtypes than for HG and FE subtypes. It is necessary to carefully select the treatment option considering endoscopic subtype and tumor size from presence of advanced histology and invasiveness.

In our study, the postprocedure bleeding and perforation rates were 8.3% and 1.6%, respectively. Postprocedure perforation was significantly more common in the PD subtype than in the other subtypes. It is probably due to have larger tumor size and higher submucosal invasion in PD subtype than the other three subtypes. However, there were no statistically significant differences between the LSTs with advanced histology and with non-advanced histology in terms of postprocedure perforation and bleeding.

Finally, we examined the significant association between advanced histology and these clinicopathological parameters after adjustments for additional confounders in the full model. Our results showed that the distal colon, larger tumor size, PD subtype, and villous component were associated with a statistically significant increased risk of advanced histology in LSTs.

In conclusion, it is clinically important to predict advanced histology before providing the appropriate treatment in LSTs. Our results indicate that the location, size, endoscopic subtype, and histologic component of the LSTs are associated with an increased risk of advanced histology. Therefore, these clinicopathological parameters may be useful in selecting therapeutic strategies in a clinical setting.

## Supporting information

S1 File Data file. (XLS)

S2 File List of all IRBs. (DOCX)
