# Supplementary material for: Clinicopathological features of laterally spreading colorectal tumors and their association with advanced histology and invasiveness: An experience from Honam province of South Korea: A Honam Association for the Study of Intestinal Diseases (HASID)
Source: PLoS One. 2017 Oct 4;12(10):e0184205. doi: 10.1371/journal.pone.0184205 (PMC5627894; doi:10.1371/journal.pone.0184205)
Supplement: S2 File — (DOCX) [file pone.0184205.s002.docx]

**Clinicopathological features of laterally spreading colorectal tumors and their association with advanced histology**

**: An experience from Honam province of South Korea**

**: A Honam Association for the Study of Intestinal Diseases (HASID)**

Dae-Seong Myung^1^, Sun-Seog Kweon^2^, Jun Lee^3^, Ik-Sang Shin^4^,

Sang-Wook Kim^4^, Geom-Seog Seo^5^, Hyun-Soo Kim^1^, and Young-Eun Joo^1^

**^1^** Department of Internal Medicine, Chonnam National University Medical School, Gwangju, Korea,

**^2^** Department of Preventive Medicine, Chonnam National University Medical School, Gwangju, Korea,

**^3^** Department of Internal Medicine, Chosun University College of Medicine, Gwangju, Korea,

**^4^** Department of Internal Medicine, Chonbuk National University Medical School, Jeonju, Korea,

**^5^** Department of Internal Medicine, Wonkwang University College of Medicine, Iksan, Korea

- Institutional Review Board of Chonnam National University Hwasun Hospital(**2013-149**),
- Institutional Review Board of Chosun University Hospital(**2014-02-005**),
- Institutional Review Board of Chonbuk National University Hospital (**2014-01-005-002**)
- Institutional Review Board of Wonkwang University Hospital(**WKUH 201401-HRE-010**).
